# Supplementary material for: Mechanisms governing the pioneering and redistribution capabilities of the non-classical pioneer PU.1
Source: Nat Commun. 2020 Jan 21;11:402. doi: 10.1038/s41467-019-13960-2 (PMC6972792; doi:10.1038/s41467-019-13960-2)
Supplement: Supplementary file 7 — Source data [file 41467_2019_13960_MOESM7_ESM.zip › Source_Data/FigureS5/FigureS5B_MotifsETS1specificPeaks/homerResults/motif8.similar.html]

motif8

## Information for motif8

T
A
C
G
T
G
C
A
G
T
A
C
G
A
C
T
T
A
G
C
G
T
A
C
G
C
T
A
G
C
T
A
A
G
T
C
G
C
A
T
A
T
G
C
  
Reverse Opposite:  

T
A
C
G
C
G
T
A
T
C
A
G
C
G
A
T
C
G
A
T
C
A
T
G
A
T
C
G
C
T
G
A
C
A
T
G
A
C
G
T
A
T
G
C
  

|  |  |
| --- | --- |
| p-value: | 1e-65 |
| log p-value: | -1.502e+02 |
| Information Content per bp: | 1.680 |
| Number of Target Sequences with motif | 219.0 |
| Percentage of Target Sequences with motif | 19.30% |
| Number of Background Sequences with motif | 330.9 |
| Percentage of Background Sequences with motif | 4.05% |
| Average Position of motif in Targets | 108.3 +/- 59.7bp |
| Average Position of motif in Background | 103.8 +/- 69.4bp |
| Strand Bias (log2 ratio + to - strand density) | -0.4 |
| Multiplicity (# of sites on avg that occur together) | 1.05 |
| Motif File: | file (matrix) reverse opposite |

### Similar de novo motifs found

|  |  |  |  |  |  |  |  |
| --- | --- | --- | --- | --- | --- | --- | --- |
| Rank | Match Score | Redundant Motif | P-value | log P-value | % of Targets | % of Background | Motif file |
| 1 | 0.757 | G T A C A C G T T G C A G T A C C G T A G T C A A T G C A G C T A G T C | 1e-55 | -128.532067 | 19.12% | 4.69% | motif file (matrix) |
| 2 | 0.764 | T C G A C A G T C T G A A G C T C T A G A C T G T G C A A C T G A C G T A G T C | 1e-16 | -37.684036 | 2.73% | 0.23% | motif file (matrix) |
